# Experimental investigation of the effect of polymer matrices on polymer fibre optic oxygen sensors and their time response characteristics using a vacuum testing chamber and a liquid flow apparatus

**DOI:** 10.1016/j.snb.2015.08.095

**Published:** 2016-01

**Authors:** Rongsheng Chen, Federico Formenti, Hanne McPeak, Andrew N. Obeid, Clive Hahn, Andrew Farmery

**Affiliations:** a Nuffield Division of Anaesthetics, Nuffield Department of Clinical Neurosciences, University of Oxford, John Radcliffe Hospital, Oxford OX3 9DU, UK; b Oxford Optronix Ltd, 19-21, Central 127, Olympic Avenue, Milton Park, Oxford OX14 4SA, UK

**Keywords:** Optical oxygen sensors, Polymer optical fibres, Time response testing, Intravascular sensor

## Abstract

Very fast sensors that are able to track rapid changes in oxygen partial pressure (PO_2_) in the gas and liquid phases are increasingly required in scientific research – particularly in the life sciences. Recent interest in monitoring very fast changes in the PO_2_ of arterial blood in some respiratory failure conditions is one such example. Previous attempts to design fast intravascular electrochemical oxygen sensors for use in physiology and medicine have failed to meet the criteria that are now required in modern investigations. However, miniature photonic devices are capable of meeting this need. In this article, we present an inexpensive polymer type fibre-optic, oxygen sensor that is two orders of magnitude faster than conventional electrochemical oxygen sensors. It is constructed with biologically inert polymer materials and is both sufficiently small and robust for direct insertion in to a human artery. The sensors were tested and evaluated in both a gas testing chamber and in a flowing liquid test system. The results showed a very fast *T*_90_ response time, typically circa 20 ms when tested in the gas phase, and circa 100 ms in flowing liquid.

## 1 Introduction

Ultrafast sensors are needed to follow rapid changes in the partial pressure of oxygen (PO_2_) in both gas and liquid phases in medicine, physiology and biology [1], [2], [3], [4]. In particular, there is an increasing interest in monitoring very fast changes in the PO_2_ of arterial blood in some respiratory conditions [5], [6], [7], [8], and only photonic devices are capable of meeting this need in terms of miniaturisation, robustness and rapid response time. Previous attempts to design electrochemical (e.g. Clark electrode) oxygen sensors for routine intravascular use in physiology or medicine have all failed to meet the required criteria [2].

Oxygen analysis in the gas phase has been traditionally achieved by electrochemical sensing, paramagnetic analysis, mass-spectrometry and more recently by fluorescence/luminescence based optical devices exploiting the Stern–Volmer relationship [9], [10]. Conventional electrochemical sensors are relatively slow, often with up to 60 s response times [11]. Although both mass spectrometry and paramagnetic devices have potentially much faster response times of 100–500 ms, they are both bulky and costly and their ultimate response times are limited by gas transport, sampling and signal processing issues. They also tend to be restricted to measurements in the gas phase only.

In contrast, optical sensors based on the well-established technique of oxygen quenching of excited luminescent dyes and optical fibre based chemical sensor (OFCS) technology overcome these limitations. In principle, a luminophore (the luminescent dye or reagent) is solubilised into a polymer matrix and coated onto the tip of an optical fibre. The immobilised luminophore is excited by an LED light source of fixed wavelength which has been transmitted through the optical fibre to the sensor tip. The (oxygen) quenched emission is then also transmitted back through the same optical fibre and measured by a sensitive photodetector. The decay rate of the luminescence from its chemically excited state is dependent upon the concentration of oxygen and can be calculated from the Stern–Volmer relationship [9], [10]. Sensors based on OFCS technology are relatively easy and inexpensive to manufacture and crucially, allow measurement in both gas and liquid phases. In addition, OFCS sensors can have lengths of many metres, do not suffer interference from electromagnetic fields (such as MRI) and can have response times of much less than 1 s [12], [13]. Typically, OFCS sensors are constructed by depositing a suitable sensing film on the blunt, distal end of a fibre using a dip-coating process which needs to be sufficiently thick to generate a strong, detectable, luminescence signal [14], [15]. OFCS sensors based on evanescent light wave transport through an optical fibre, have also been reported [16], [17]. In such cases, the sensing film (which has a low refractive index relative to the fibre core) is coated along the sides of the optical fibre. The evanescent excitation light (which represents only a fraction of the overall excitation energy) penetrates the surrounding, thin-coated film to excite the embedded luminophore. With this technique, the light interaction length needs to be typically several centimetres along the fibre in order to generate enough detectable luminescence, because the luminescent excitation efficiency is relatively poor.

To improve the overall signal to noise (S/N) ratio of the detectable luminescent light, several reported studies have focused on using large-diameter, polymer type optical fibres (e.g. PMMA) for oxygen sensing [18], [19], which due to their larger surface area, provide improved S/N performance and are also suitable for invasive medical applications due to their inherent robustness when compared with standard silica fibres. Tapered-tip polymer fibre based oxygen sensors have also been investigated [20], [21]. In these studies, a tapered-style sensing tip was used to achieve high luminescent signal level and increased S/N performance of the sensing system. However, these sensors were only evaluated in a gaseous environment and the results showed the sensors to be both relatively insensitive and with slow response times to changes in PO_2_. We propose this may have been due to the low oxygen permeability of the type of sensing matrix used in the studies and the possibility that oxygen may have diffused and become trapped in the polymer fibre itself, thereby creating a ‘reservoir effect’ which would effectively limit the overall response time of the sensor.

**Fig. 1:**
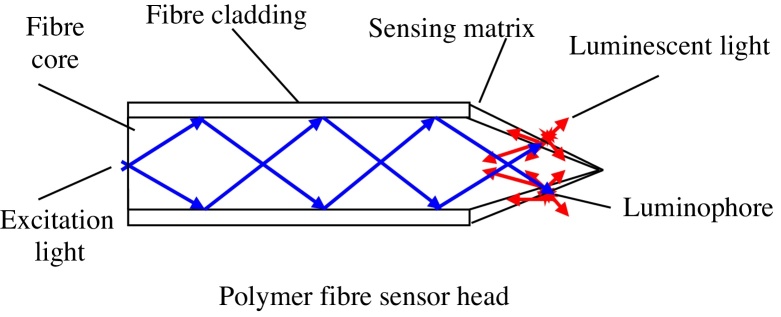
Schematic illustration of the tapered-tip design.

Here, we present a small, sensitive and ultra-fast response time oxygen sensor also constructed from polymer type optical fibre. Fig. 1 shows the schematic of a polymer fibre optic sensor head.

We investigated the influence of using different polymer matrix materials in our previous study [1] in which three different polymer materials were compared in terms of oxygen (PO_2_) sensitivity and *T*_90_ time response in a series of sensor tests in the gaseous phase. Our experimental results revealed that oxygen sensors constructed with a PPMA based luminophore matrix provided higher sensitivity and faster time responses than those made with either PMMA or PEMA matrices in our PO_2_ range of interest (approx. 5–30 kPa).

## 2 Principle and sensor fabrication

### 2.1 Principle

The optical oxygen sensor presented here is based on luminescence quenching by oxygen of a fluorophore immobilised in the matrix material. Assuming dynamic quenching only, the luminescence intensity and luminescence life time are directly related to the oxygen concentration according to the Stern–Volmer relation [9]:

(1) $$I_{0}/I=\tau_{0}/\tau =1+\text{Ksv}[{\text{O}}_{2}]=1+k\tau_{0}[{\text{O}}_{2}]$$

where *I*_0_ and *I* are the intensities of the luminescence in the absence and presence of oxygen respectively; *τ*_0_ and *τ* are the lifetime of the excited state luminescence in the absence and presence of oxygen respectively; Ksv is the Stern–Volmer quenching constant; [O_2_] is the oxygen concentration; *k* is the bimolecular rate that describes the efficiency of the collision between the luminophore and oxygen molecules. Under ideal conditions, the plot of (*I*_0_/*I* − 1) or (*τ*_0_/*τ* − 1) against [O_2_] is linear with a slope equal to Ksv, and can be used for simple sensor calibration.

### 2.2 The polymers

**Fig. 2:**
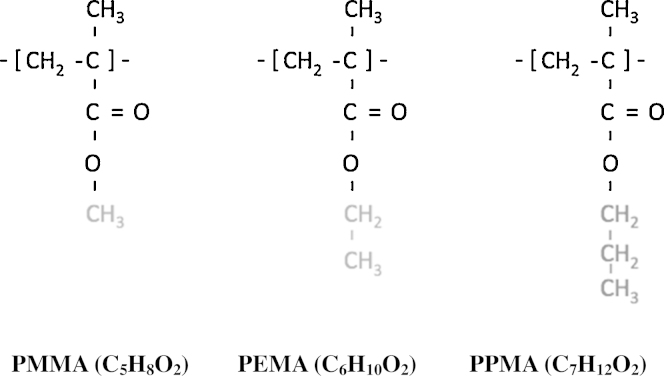
Molecular structures of the different polymer matrices.

To examine the influence of the oxygen diffusivity on sensor time response, the oxygen quenching luminophore, (PtOEP, a Platinum (II) complex) was first immobilised and then tested in a series of several different acrylate type polymers. Acrylate polymers are generally considered biologically inert and therefore potentially safe for medical applications [22]. In most polymer materials, the larger pendant groups prevent the polymer chains closing and packing together, so polymers with larger pendant groups will tend to have higher oxygen solubility and higher diffusion coefficients [23], [24]. In this study we tested three acrylate polymers: poly(methyl methacrylate) (PMMA), poly(ethyl methacrylate) (PEMA) and poly(propyl methacrylate) (PPMA) as matrix materials to immobilise the Platinum (II) complex. These three polymers have similar main structures but different pendant group sizes (see Fig. 2) and hence different oxygen permeability. Theoretically, a sensor using PPMA as the matrix should have both higher sensitivity and a faster time response [1].

### 2.3 Sensor preparation

Matrix polymer materials of poly(methyl methacrylate) PMMA, poly(ethyl methacrylate) PEMA, and poly(propyl methacrylate) PPMA and the solvent of dichloromethane were obtained from Sigma–Aldrich (USA). The Platinum-Octaethyl-Porphyrin (PtOEP) luminophore was purchased from Porphyrin (USA). All reagents were analytical grade and used as received. Three luminophore-doped polymer sensing solutions were prepared by mixing and dissolving 1 mg PtOEP and 100 mg each type of polymer material in 2 ml dichloromethane respectively. These mixtures were stirred to ensure complete dissolution of each polymer and luminophore. Prior to dip-coating the thin film, a 5 mm distal section of polymer fibre with overall length of 2 m (Toray Industries, INC) was heated using a heat gun set at 150 °C. A tapered tip was formed by pulling the fibre until it broke into two parts. The tapered tip of the polymer fibre was cleaned with a solution of isopropyl alcohol and allowed to dry for 10 minutes. The oxygen sensing tip was formed by dip-coating a 1 mm section of the tapered fibre tip into the luminophore-doped polymer solution and withdrawing it slowly while the solvent quickly evaporated. The sensing tip was allowed to dry at room temperature for a further 24 h.

## 3 Experimental results

### 3.1 Experimental setup

In our experiments, the oxygen sensing system consisted of a fibre-optic oxygen sensor and a Y type optical fibre coupler. The excitation light was provided by a wideband LED light source (LS-450, Ocean Optics Inc, USA) with a peak wavelength of 450 nm, which covers the absorption spectrum of the luminophore. This excitation light was launched into the sensor head via the one arm of the optical fibre coupler and the luminescent light emitted from the sensor tip was collected through the other arm of optical fibre coupler. In the gas phase tests, the luminescence output from the fibre was detected and measured as luminescence intensity using a spectrophotometer (USB 2000 Ocean Optics Inc, USA).

In the liquid tests, luminescence phase detection was used. In this case, the sensor tip was inserted into the test circuit [25] and the modulated UV LED broadband light, which is integrated in a luminescence phase measurement system (NeoFox, Ocean Optics Inc, USA), was used to excite the luminophore immobilised in the sensing matrix. The decay lifetime of the excited luminescence light from the sensor tip was calculated and measured by the NeoFox. Water was used as the test liquid.

In order to evaluate sensor time response in the gas phase, a modification of a gas pressure chamber test system was used [26]. The system comprises an aluminium cylindrical test chamber, with internal diameter and length of 2 cm and 10 cm respectively, packed with steel ball bearings to reduce the cylinder gas volume. The tip of the optical sensor was inserted into the test chamber via a sealing grommet. To provide a swift oxygen partial pressure change, a gas cylinder containing room air was connected to the test chamber via a pressure reducing valve and a buffer chamber, with the pressure set at 1.0 bar. The test chamber was also connected to a second buffer chamber that was continuously evacuated by a vacuum pump (MZ 2C NT, Vacuubrand GMBH, Wertheim, Germany). The chamber also had a controlled leak to atmosphere so that the ambient pressure in the chamber could be reduced to any preset level below atmospheric pressure – and hence the chamber (PO_2_) reduced to below 21 kPa. Using solenoid valves to switch the test chamber to either of the two buffer chambers, the total ambient pressure in the test chamber was quickly changed between 1.0 bar and any pressure below this. Thus, PO_2_ in the test chamber could be switched swiftly between any preset level between 21 and 0 (vacuum) kPa. The dynamic change in total pressure was measured by a Honeywell piezo resistive pressure sensor with a time response of 1 ms (RS Components Ltd, UK). Adjusting the leak to keep the chamber above vacuum provided the smallest chamber time constant. The head of the sensor probe was inserted into the test chamber and the response time of the sensor probe was tested in response the above changes in PO_2_. The response signal of the peak wavelength (645 nm) of the luminescent emission from the oxygen sensor head was measured using a USB 2000 spectrometer with 20 or 50 ms integration times. This also sets a limit on the time resolution which our apparatus could determine.

A flowing liquid test circuit was used to generate rapid PO_2_ changes in the liquid phase. Full technical details of this system have been previously presented [25]. Briefly, two standard medical paediatric oxygenators (Medos Hilite 1000LT, Medos Medizintechnik AG, Stolberg, Germany) were arranged to provide two parallel and independent extracorporeal circuits, where the PO_2_ in the test liquid, in this case water, can be varied from 0 kPa to 100 kPa for sensor sensitivity testing; or the two circuits maintained at 5 kPa and 30 kPa PO_2_ level for sensor time response testing. The PO_2_ reference values were confirmed through a blood gas analysis (ABL710 Blood Gas Analyser, Radiometer, Copenhagen, Denmark) for sensor calibration purposes and for monitoring before each experiment. Two peristaltic pumps maintained the water flow through the two parallel circuits. The water temperature was maintained at 37 °C by passing water from a separate heated circuit (Grant Instruments, Cambridge, UK) through the two oxygenators. The water temperature was continuously monitored with a thermocouple (TES130, TES Electrical Electronic Corp., Taipei, Taiwan). Flow from either circuit was diverted alternately towards the sensor being tested by means of computer-controlled rapid switchover solenoid valves that exposed the sensor to abrupt liquid PO_2_ changes. The flow system cross-over response-time (*T*_10–90%_) was calculated to be circa 50 ms [25].

### 3.2 Gas chamber sensor time response results

**Fig. 3:**
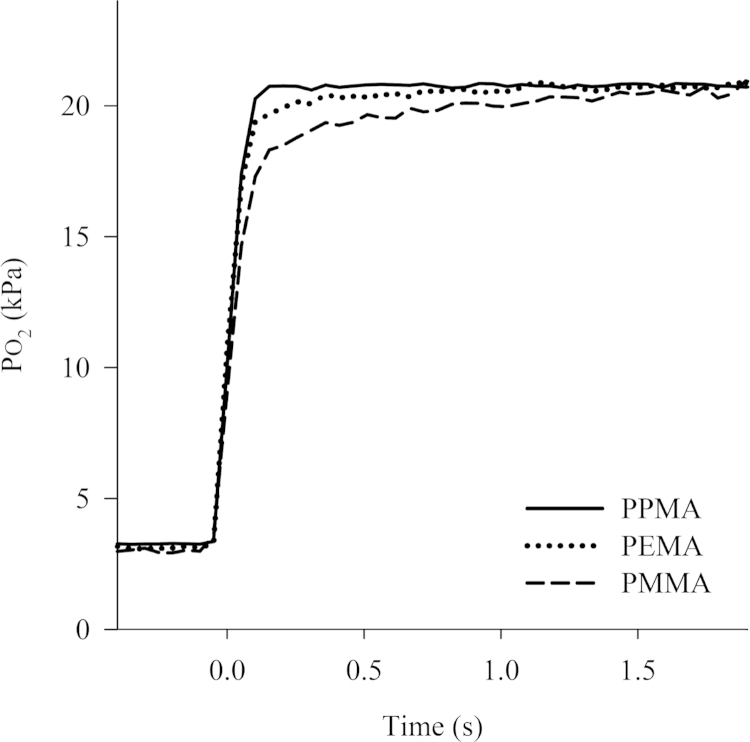
Typical plots of PO_2_ versus time for fibre optic oxygen sensors manufactured from three different polymer matrices to a step change in PO_2_ from 3 kPa to 21 kPa.

Fig. 3 shows the PO_2_ signal versus time responses for oxygen sensors constructed using the three different acrylate polymers. In each case, a PO_2_ step change from 3 kPa to 21 kPa was evoked in test chamber and the spectrophotometer integration time set to 50 ms in order to improve the S/N ratio.

These results show that sensors made from polymers with different size pendant groups have different response times. Oxygen sensors constructed with the PPMA matrix exhibited higher sensitivity and a faster time response than those sensors constructed with PMMA or PEMA matrices.

**Fig. 4:**
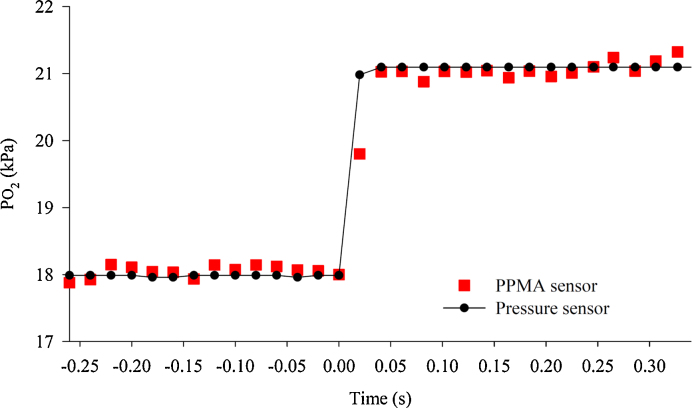
Plot of the change in oxygen partial pressure versus time for a PPMA oxygen sensor (red squares), and the piezo pressure sensor (filled circles), when the PO_2_ was rapidly switched from 18 kPa to 21 kPa. The sampling rate was 20 ms. (For interpretation of the references to colour in this figure legend, the reader is referred to the web version of this article.)

Fig. 4 shows a typical time response of the PPMA sensor for a step change in PO_2_; in this instance, from 18 kPa to 21 kPa. Here, the integration time (or averaging) of the spectrophotometer was reduced to 20 ms (in order to extract the maximal time response), but at the expense of an increase in noise on the sensor output signal. As a reference, the change in PO_2_ based on the total chamber pressure change measured with the piezo resistive pressure sensor, is also shown. This reference (filled circles, shown in Fig. 4) shows the true overall dynamic time constant (*T*_10–90%._) of the pressure chamber itself to be less than 20 ms. The corresponding optical sensor response (red squares) closely overlaps that of the piezo pressure transducer response, and so our data cannot distinguish between the time responses of the two sensors. From the near inseparability of the oxygen pressure signal and the total pressure signal (whose response time is 1 ms), we conclude that the time response of the oxygen sensor is of the order of a few milliseconds.

### 3.3 Sensor results in flowing liquid

**Fig. 5:**
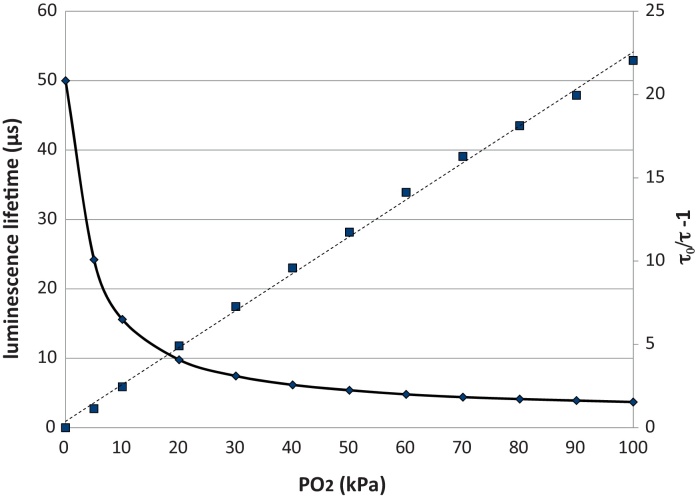
Change in luminescence lifetime versus PO_2_ (solid line) and Stern–Volmer plots (dashed line) for the PPMA oxygen sensor in the liquid test circuit. Results are for water at 37 °C.

Fig. 5 shows the change of luminescence lifetime versus steady-state oxygen partial pressures for the PPMA oxygen sensor in the water test circuit at room temperature. Fig. 5 also shows the Stern–Volmer plot for the same (representing sensor sensitivity), which shows a linear relationship, with a sensitivity (Ksv) of around 23.

**Fig. 6:**
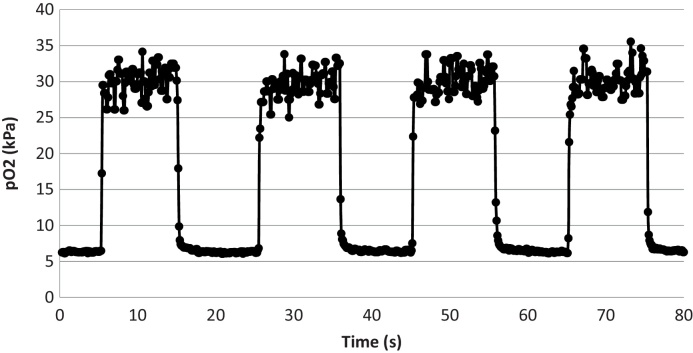
PPMA sensor response to PO_2_ step changes 5 kPa to 30 kPa, and 30 kPa to 5 kPa, in the liquid test circuit. The sensor was 37 days post manufacture. Results are for water at room temperature. Sampling points are 100 ms. A 3 sample median filter has been applied.

Fig. 6 shows the PPMA sensor response to PO_2_ step changes from 5 kPa to 30 kPa, and 30 kPa to 5 kPa, in the liquid test circuit on day 37 post manufacture. It is clear that the step-change has reached its full value within c. 100 ms. Since we have previously shown that the time of switch-over of the two flowing liquids is about 50 ms [25], we can expect the PO_2_ time response of the sensor to be better than 100 ms in liquid.

## 4 Discussion

Our results show that the performance of luminescence based fibre optic sensors can be improved by using optimised polymers for the luminophore matrix and that the time response of such sensors is slower in the liquid phase when compared to the gas phase. This could be due to the fact that oxygen diffuses more slowly between water and the polymer matrix than between gas and the matrix due to the larger molar concentration of oxygen in the latter for the same PO_2_. However, it should be noted that the response time of the liquid test rig itself is finite (of the order of 50 ms [25]) and so this is responsible for some part of the measured sensor response time (*T*_10–90_ circa 100 ms).

This work has focussed solely on membrane covered *polymer* (as opposed to silica) fibres, designed for PO_2_ sensing in breath analysis [1] or in flowing liquid, such as in arterial blood [4], [27]. We acknowledge that there are other much faster submillisecond oxygen sensing devices already described for areodynamics and acoustic investigations and for biological analysis (e.g. [28], [29], [30]), but these devices cannot be inserted into flowing blood in a human artery. Our polymer fibre based devices are slower than those designed for aerodynamic purposes, but are nonetheless fast enough (*T*_10–90_ circa 100 ms) for clinical use and to investigate physiological phenonema. To our knowledge, there is no device presently available that allows us compare our sensor performance in a flowing liquid. We have therefore relied on a vacuum chamber (as have other authors [29], [31]) and a flowing liquid test rig [25] to provide our near-gold standard.

One potential problem with this type of sensor is hysteresis caused by asymmetry in the kinetics of oxygen diffusion into and out of the polymer matrix. If the polymer fibre itself were to act as an oxygen reservoir, one might expect to see a slow response of the sensor to a step *drop* in PO_2_ in the medium being sensed. This is due to continued diffusion of oxygen from the reservoir, across the sensing matrix and into the sensed medium. Our results demonstrate that this is not the case for steps between 30 and 5 kPa.

Our results also show that the time-response of the sensors is rapid after a period of at least one month after manufacture. Indeed, the time-response on day 37 post-manufacture is rapid enough for our purposes in physiological measurement. This finding supports the supposition that no significant physical or chemical degradation or luminophore loss has occured over this period.

## 5 Conclusions

In summary, we have demonstrated that a fibre optic oxygen sensor with an optimised polymer matrix can be used for measuring physiologically rapid changes in oxygen partial pressure in both gas and liquid phases. The maximum sensitivity factor of the sensors ($I_{{\text{N}}_{2}}/I_{{\text{O}}_{2}}-1$) to changes in oxygen over the range 0–100% was approximately 23 with a maximum response time of a few ms in the gas phase. The ability to determine this more precisely is limited by the time resolution of both the gas phase and liquid flow test apparatuses. The true sensor response time may be even faster than we speculate, but we are unable to resolve this. These polymer based optical fibre sensors are small, robust and fast enough to be used in biological and medical and other applications where the PO_2_ is changing rapidly.
